# Apoptosis-related gene expression in glioblastoma (LN-18) and medulloblastoma (Daoy) cell lines

**DOI:** 10.1007/s13577-011-0029-9

**Published:** 2013-09-15

**Authors:** Iwona Wybranska, Anna Polus, Magdalena Mikolajczyk, Anna Knapp, Agnieszka Sliwa, Barbara Zapala, Teresa Staszel, Aldona Dembinska-Kiec

**Affiliations:** 1 Department of Genetic Diagnostics and Nutrigenomics, Chair of Clinical Biochemistry, The Jagiellonian University, Medical College, Kraków, Poland; 2 Department of Clinical Biochemistry, Chair of Clinical Biochemistry, The Jagiellonian University, Medical College, Kraków, Poland

**Keywords:** Apoptosis, Models for brain cell research, LN18, Daoy, Inflammation, Brain cancer cell lines

## Abstract

The expression of apoptosis genes in a commercial pre-designed low-density array from Applied Biosystems was evaluated in two human brain cancer cell models, LN-18 and Daoy (HTB-186™) in comparison to the reference human primary endothelial cells under basic conditions. Analysis of the gene expression in the cancer cell lines compared to the normal control revealed features reflecting anti-apoptotic and inflammatory characteristics of the former. There was an overall downregulation of apoptosis-stimulating genes in both cancer cell lines, along with an upregulation of certain apoptosis inhibitors. A number of genes demonstrated statistically significant changes in their expressions, including BAX (BCL2-associated X protein); the CARD4/NLR family, CARD domain containing 4; CASP10 (caspase 10, apoptosis-related cysteine peptidase); DAP1 (death-associated protein kinase 1), and BIRC5 (baculoviral IAP repeat-containing 5). Anti-apoptotic potential in both cell lines was demonstrated by changes in the Bax:Bcl-2 ratio and downregulation of the APAF1 gene in LN18 cells. There was also significant downregulation of extrinsic signals and the TNF/FADD/inflammatory cascade, and upregulation of caspase inhibitors (IAPs). These results provided a novel molecular characterization of important human cancer cell lines, which might provide a useful research tool for investigating the experimental model of the CNS cell.

## Introduction

Human cancer research heavily relies on molecular biology, which, among other methods, includes controlled studies in culture cell lines. Even though typical commercially available cell lines often differ from normal and cancer cells, difficult access to human tumor and/or human normal tissue makes them useful and popular experimental models. Two standard tumor brain cell lines from the American Type Culture Collection, LN18, derived from glial cells (supportive tissue of the brain) and Daoy, derived from the neural stem cell precursors, are widely used in studies that employ experimental models of brain neoplastic disease.

Medulloblastoma, the most common malignant brain tumor in children, is a primitive neuroectodermal tumor arising in the cerebellum. The aggressive clinical behavior of the tumor along with the cognitive and endocrinological long-term side effects of current therapies mean that both the development of prognostic indicators for disease stratification and the identification of new therapeutic targets represent major goals. Current understanding of the molecular biology of medulloblastoma is limited. Cytogenetic studies have described consistent chromosomal aberrations, but molecular genetic studies have identified specific genetic and epigenetic abnormalities in only a small proportion of those tumors (reviewed in [1–3]). The Daoy cell line was established in 1985 by P.F. Jacobsen of the Royal Perth Hospital in Western Australia. The line was derived from the biopsy material taken from a tumor in the posterior fossa of a four-year-old boy. Although the original tumor presented characteristics of both neuronal and glial phenotypes, these were not retained by the Daoy cell line [4].

Gliomas are the most frequent and lethal cancers originating in the central nervous system in adults. The LN-18 cell line was established in 1976 from the cells of right temporal lobe glioma. The cells are poorly differentiated [4–7] and negative for glial fibrillary acidic proteins and S100 (S-100) protein [5]. They exhibit mutated p53 (TP53) and possible homozygous deletions in the p16 and p14ARF tumor suppressor genes, and have a wild-type PTEN gene [6]. Stimulation of the cells with Fas ligand leads to apoptotic cell death within 16 h. Overexpression of Bcl-2 protects these cells from Fas ligand-induced cell death. This cell line is frequently used in apoptosis studies [8].

Apoptosis can result from the activation of multiple pathways that differ according to tissue type and pathological condition. They have been identified and classified into two main types: extrinsic and intrinsic pathways. Both pathways converge finally at the level of the activation of caspases, the effector molecules in most forms of cell death. The extrinsic pathways include death-receptor and survival-factor withdrawal pathways. The first is activated by the stimulation of certain membrane receptors such as TNF-alpha and Fas receptors, while the latter involves the activation of c-Jun and JNK. Research efforts for the past two decades have revealed the existence of a superfamily of 18 genes encoding 19 TNF ligands that signal through 30 receptors. One ligand has been found to bind to as many as five different receptors. This superfamily is associated with several disease conditions, including acute and chronic inflammatory and genetic diseases [10–12]. The TNF family and its corresponding receptors play important roles in cell death or survival, proliferation, and maturation. A specific characteristic of all members of the TNF superfamily is that, through the recruitment of TNF receptor-associated factors (TRAFs), they all activate NFkB, a transcription factor that causes the suppression of apoptosis, cell survival, and proliferation. Some TNF ligands can activate both apoptosis and anti-apoptosis cell survival pathways under certain conditions. The balance between these two types of pathways gives this superfamily the ability to act as a switch for cell death or survival [11, 13].

The Bcl2 family consists of approximately 15 proteins. The family can be divided into anti-apoptotic and pro-apoptotic subgroups that share one or more Bcl2 homology domains (BH1–BH4). The anti-apoptotic members, which include Bcl2, Bcl-xL (the long isoform encoded by Bcl2l1), Bcl-W (Bcl2l2), Mcl1, and A1/Bfl1 (Bcl2a1), all share BH1 and BH2 domains. They are thought to exert their effects by stabilizing the mitochondrial membrane potential and preventing the release of cytochrome c and apoptosis inducing factor (AIF). Bcl2 and Bcl-xL can block cell death caused by a wide variety of apoptotic stimuli, such as chemotherapeutic drugs, ultraviolet radiation, heat shock, free radicals, calcium ions, TNF, and interleukin-3 [14–16]. The pro-apoptotic members, which include Bax, Bak, Bok, Bid, Bad, Bcl-xS (the short isoform encoded by Bcl2l1), Bim (Bcl2l11), Bik, Blk, and Hrk, have the BH3 domain, which is necessary for their pro-apoptotic effect. Interactions between anti-apoptotic and pro-apoptotic members of the Bcl2 family determine the sensitivity of a cell to apoptosis [17–20]. While Bcl-xL (Bcl2l1) appears to be the most dominant anti-apoptotic factor in the developing central nervous system and adult neurons, both Bcl2 and Bcl-xL are expressed in embryonic neurons [21]. Bcl-xL expression levels have been found to be high prior to birth, undetectable in the neonatal tissue, low in young adult brain, and elevated again in the aged human brain [22], while the overexpression of Bcl2 prevents cell death.

The release of cytochrome c into the cytoplasm results in the activation of an apoptosome complex composed of APAF1, cytochrome c, and deoxy-ATP. This complex stimulates caspases and initiates cell death by breaking up different cell proteins and DNA fragments through CAD (caspase activated DNase)-mediated degradation.

Caspases are a family of cysteine proteases that play a critical role in human cell apoptosis, the activation of cytokines, and the induction of inflammation. To date, 11 human caspases have been identified. They are divided into two main classes: upstream or initiator caspases (Casp1, 2, 4, 5, 8, 9, 10, 12, 13), and downstream or effector caspases (Casp3, 6, 7). Upstream caspases are responsible for caspase activation or regulation of inflammatory processes. They share long N-terminal pro-domains, caspase recruitment domains (CARDs), or death effector domains (DEDs). Downstream caspases cause the actual destruction of the cell, and they tend to have short or absent domains [23]. Another classification categorizes caspases into interleukin-1β converting enzyme (ICE) caspases (human Casp1, 4, 5, 13, 14 and mouse casp11 and 12) and CED-3 caspases (Casp 3, 6, 7, 8, 9 and 10) [24, 25]. Overexpression of either caspase 1 or 11 induces apoptosis, which can be prevented by Bcl2. Unlike caspase 1, caspase 11 mediates the lipopolysaccharide (LPS) neurotoxicity [26] and regulates cell migration [27]. In Fas- and TNF-mediated apoptosis, the Casp1 protease family seems to act downstream of caspase 8 and caspase 10, and upstream to the caspase 3 protease family [28]. Human caspase 4 may also be involved in a different apoptotic pathway, since it has been activated by agents that cause stress of the endoplasmic reticulum (ER), inducing the activation of caspase 3 [25]. The proteolytic activity of caspases is tightly controlled by the inhibitors of apoptosis protein family (BIRC/IAP), which have evolved to protect cells from unwanted self-execution through chance activation of the death cascade.

Our study is based on the fact that patterns of expression of known genes can reveal novel phenotypic aspects of the cells and tissues studied. The predefined apoptosis gene signature was studied in Daoy medulloblastoma cells, LN18 glioblastoma cells, and primary human umbilical vein endothelial cells (HUVEC) using commercial 384-well format microfluidic cards (TLDA TaqMan^®^ human apoptosis array from Applied Biosystems) based on the RT-PCR reaction.

## Methods

### Cell culture

The human glioblastoma cell line originating from right temporal lobe cells (LN18) (ATCC, CRL-2610) cultured in Dulbecco’s modified Eagle’s medium (ATCC) with 5% foetal bovine serum, FBS (Clonetics), and human medulloblastoma cells (Daoy) (ATCC, HTB-186) cultured in Eagle’s minimum essential medium (ATCC, 30-2003) with 10% FBS (Clonetics) were used. Cells were cultured at 37°C with 95% humidity and 5% CO_2_.

Additionally, as a reference, primary human umbilical vein endothelial cells (HUVEC) were isolated from the umbilical cords using collagenase digestion according to Jaffe et al. [9]. The HUVECs were cultured in EBM (EGM Bullet Kit, Clonetics) with supplements: hEGF (10 ng/ml), hydrocortisone (1 ng/ml), bovine brain extract (12 μg/ml), antibiotics: gentamicin (50 μg/ml) and amphotericin B (50 ng/ml), and 10% FBS (Clonetics). HUVECs up to the fifth passage were used for the experiments. Cells were cultured at 37°C with 95% humidity and 5% CO2.

### The microarray study (TaqMan^®^ human apoptosis array: Applied Biosystems TLDA)

The regulation of gene expression was studied in triplicate (three different experiments on three separate TLDA plates), using predesigned 384-well microfluidic cards (TLDA TaqMan^®^ human apoptosis array from Applied Biosystems) based on the RT-PCR reaction.

### RNA extraction and reverse transcription

Total RNA from the experimental and control cells was isolated by a TRIzol^®^ Plus RNA purification system (cat. no. 12183-555, Invitrogen). The RNA isolation quality was checked by NanoDrop^®^. Five micrograms of total RNA were reverse transcribed in a total volume of 15 μl using the First-Strand DNA synthesis kit according to the manufacturer’s guidelines (cat. no. 4368814, Applied Biosystems).

### The qRT-PCR based TaqMan low-density array (TLDA)

The predesigned 384-well microfluidic cards with eight sample loading ports were used (TLDA TaqMan^®^ human apoptosis array, Applied Biosystems, cat. no. 4378701). The TLDA cards were configured into four identical 96-gene sets measured in triplicate. The TaqMan low-density array human apoptosis panel contained assays for 93 human genes in addition to three endogenous controls (18S, ACTB, GAPDH). The primer sets (TaqMan Gene Expression Assay, Applied Biosystems) were selected so that they generated amplicons that were no longer than 100 bp (mean 74 bp). The 93 genes were categorized into multiple target classes or pathways including intrinsic, extrinsic, regulatory, and execution apoptosis traits. A reaction mixture with cDNA template (100 ng) and an equal volume of TaqMan^®^ universal master mix (Applied Biosystems) was immediately loaded into each line of the TLDA microfluidic card. Each card was spun twice at 1,200 rpm for 1 min each time to distribute the PCR mix into the wells of the card, before it was sealed and loaded into the ABI 7900HT fast real-time PCR system. The thermal cycle conditions were as follows: 2 min at 50°C, 10 min at 94.5°C, 30 s at 97°C, and 1 min at 59.7°C for 40 cycles.

### Array normalization and selection of differentially expressed genes from microarray data

The arithmetic standard normalization procedures recommended by the Data Assist software for microarray data were followed. In brief, data transformation was corrected for the signal from the three endogenous controls (18S, ACTB, GAPDH). Per card (mean) normalization accounted for the variability of each card by dividing all of the measurements on each card by the value of the 50th percentile. Per gene normalization accounted for the variability between probe sets for the three reporter genes.

The threshold cycle, Ct, was automatically assigned by the SDS2.2 software package (Applied Biosystems). Relative quantities (RQ) were determined using the equation RQ = 2 − ΔΔCt. All data were generated in triplicate (different TLDA plates) and expressed as the mean ± SD. Differentially expressed genes were selected from the normalized data using a procedure known as significance analysis of microarrays, as installed in the SDS2.3 software package (Applied Biosystems). Genes were considered to be significant when their average fold change (FC) was ≤−1.5 or ≥1.5, and statistically significant when the corresponding *p* value was ≤0.05. Two software programs were used to analyze the data, namely SDS RQ Manager 1.2 and DataAssist v.2.2 software (Applied Biosystems).

MetaCore™ software (from GeneGo) was used to perform pathway analysis of the differentially expressed genes.

## Results

The expressions of the 93 genes that constitute the most significant apoptosis and apoptosis signal pathway-related genes were studied in the LN18 and Daoy cell lines using TaqMan low-density arrays prepared as predesigned 384-well microfluidic cards with eight sample loading ports (TLDA TaqMan^®^ human apoptosis array, Applied Biosystems, cat. no. 4378701). Three internal controls, which included glyceraldehyde-3-phosphate dehydrogenase (GAPDH), 18S rRNA and beta-actin (ACTB) were used for data normalization. Every cell line was evaluated in triplicate, in three independent cell cultures. The results were expressed as the mean values of the three experiments.

### Evaluation of differential gene expression by low-density arrays

**Fig. 1:**
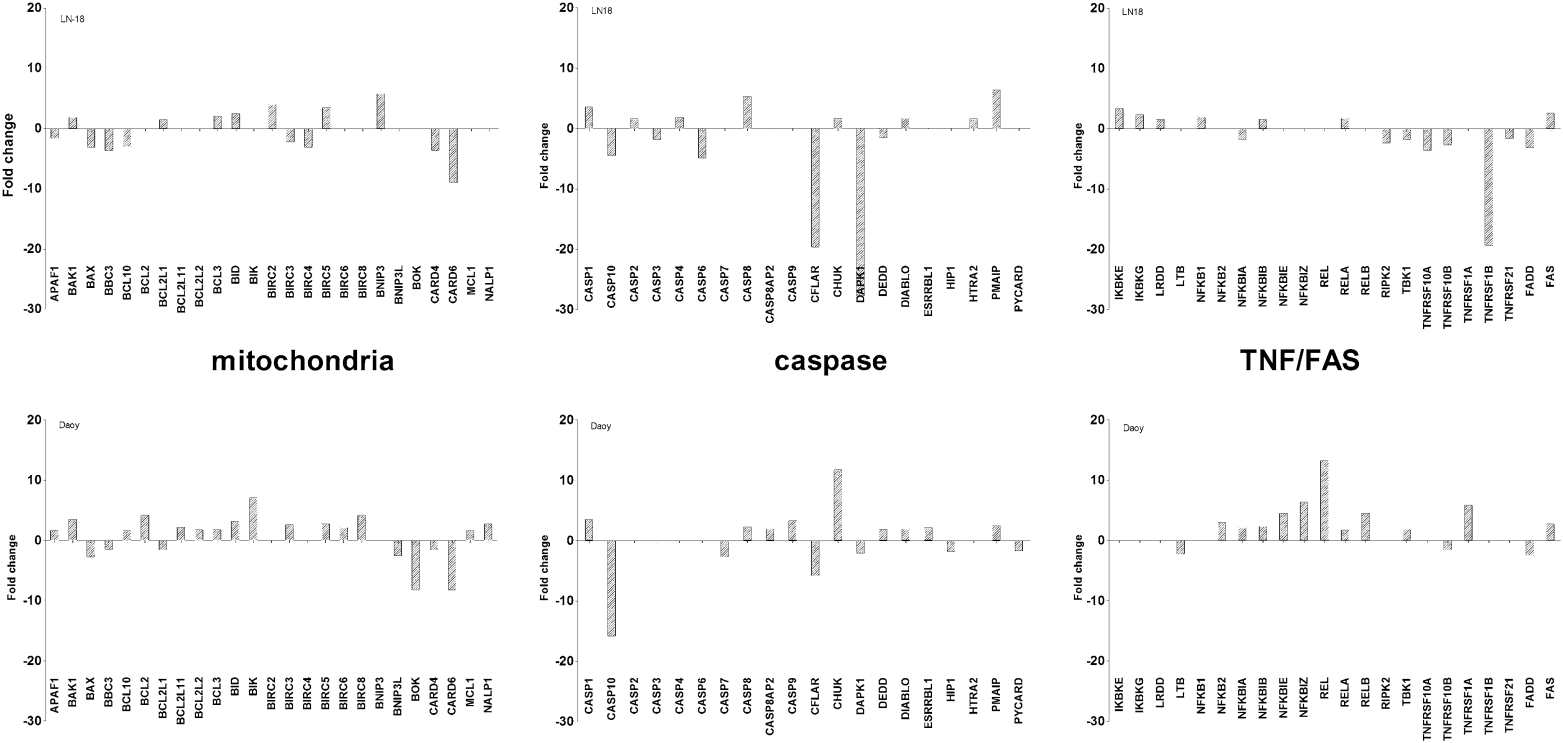
List of the genes for which expression in LN18 and Daoy cells was significantly different from the control HUVEC cells. The genes are divided into indicated apoptosis pathways: the TNF receptor pathway, NKkB signaling pathway, Bcl-2 family-regulated pathway, CARD family, caspases, IAP family, and others. Differences in gene expression are presented as fold changes (compared to reference HUVECs), accompanied by corresponding *p* values (according to [36])

Table 1 and Fig. 1 show the mean fold change (FC) in expression of the particular gene relative to the mean of the control non-cancer group (HUVECs) as a reference. ANOVA analysis with Bonferroni correction was used to determine statistical significance (*p*).

**Table 1:** Differentially expressed genes in the LN18 and Daoy cell lines. HUVEC cells were used as reference

Gene name	Ensembl gene number	LN18 (fold change)	*p*	Daoy (fold change)	*p*	Gene description
TNF receptor pathway						
TNFRSF1A	ENSG00000067182			5.85	0.4526	Tumor necrosis factor receptor superfamily, member 1A
TNFRSF1B	ENSG00000028137	−19.42	0.0159	−1.56	0.4410	Tumor necrosis factor receptor superfamily, member 1B
TNFRSF10B	ENSG00000120889	−2.73	0.0179			Tumor necrosis factor receptor superfamily, member 10b
TNFRSF10A	ENSG00000104689	−3.62	0.2720			Tumor necrosis factor receptor superfamily, member 10a
TNFRSF21	ENSG00000146072	−1.68	0.1542			Tumor necrosis factor receptor superfamily, member 21
RIPK2	ENSG00000104312	−2.37	0.4262			Receptor-interacting serine-threonine kinase 2
FAS	ENSG00000026103	2.57	0.2923	2.70	0.0736	Fas (TNF receptor superfamily, member 6)
LRDD	ENSG00000177595	1.53	0.4948			Leucine-rich repeats and death domain containing
FADD	ENSG00000168040	−3.13	0.4149	−2.46	0.4763	Fas (TNFRSF6)-associated via death domain
NF-κB signaling pathway						
IKBKG	ENSG00000073009	2.30	0.0012			Inhibitor of kappa light polypeptide gene enhancer in B-cells, kinase gamma
IKBKE	ENSG00000143466	3.30	0.4973	4.53	0.0665	Inhibitor of kappa light polypeptide gene enhancer in B-cells, kinase epsilon
NFKB1	ENSG00000109320	1.95	0.5487			Nuclear factor of kappa light polypeptide gene enhancer in B-cells 1
NFKB2	ENSG00000077150			2.95	0.1673	Nuclear factor of kappa light polypeptide gene enhancer in B-cells 2 (p49/p100)
TBK1	ENSG00000183735	−1.80	0.0835	1.90	0.0670	TANK-binding kinase 1
CHUK	ENSG00000213341	1.64	0.0817	11.71	0.3812	Conserved helix–loop–helix ubiquitous kinase
REL	ENSG00000162924			13.19	0.4201	v-rel reticuloendotheliosis viral oncogene homolog (avian)
RELA	ENSG00000173039	1.71	0.3595	1.73	0.4992	v-rel reticuloendotheliosis viral oncogene homolog A (avian)
RELB	ENSG00000104856			4.51	0.0148	v-rel reticuloendotheliosis viral oncogene homolog B
Kappa light polypeptide gene enhancer in B-cells 3, p65						
NFKBIA	ENSG00000100906	−1.86	0.0483	2.11	0.0013	Nuclear factor of kappa light polypeptide gene enhancer in B-cells inhibitor, alpha
NFKBIB	ENSG00000104825	1.58	0.3221	2.33	0.0192	Nuclear factor of kappa light polypeptide gene enhancer in B-cells inhibitor, beta
NFKBIZ	ENSG00000144802			6.33	0.1677	Nuclear factor of kappa light polypeptide gene enhancer in B-cells inhibitor, zeta
LTB	ENSG00000206437			−2.23	0.0000	Lymphotoxin beta (TNF superfamily, member 3)
Bcl-2 family-regulated pathway						
BCL10	ENSG00000142867	−2.94	0.0956	1.55	0.4350	B-cell CLL/lymphoma 10; hypothetical LOC646626
BCL2	ENSG00000171791			4.15	0.2941	B-cell CLL/lymphoma 2
BCL2L11	ENSG00000153094	1.51	0.2924	2.14	0.0856	BCL2-like 11 (apoptosis facilitator)
BCL2L2	ENSG00000129473			1.76	0.0521	BCL2-like 2
BCL2L1	ENSG00000171552			−1.58	0.3776	BCL2-like 1
BIK	ENSG00000100290			7.02	0.0006	BCL2-interacting killer (apoptosis-inducing)
PMAIP (NOXA)	ENSG00000141682	6.38	0.0906	2.44	0.1623	Phorbol-12-myristate-13-acetate-induced protein 1
BAX	ENSG00000087088	−3.06	0.0001	−2.78	0.0003	BCL2-associated X protein
BAK1	ENSG00000030110	1.89	0.0658	3.48	0.3774	BCL2-antagonist/killer 1; BCL2-like 7 pseudogene 1
BCL3	ENSG00000069399	2.04	0.3101	1.74	0.0769	B-cell CLL/lymphoma 3
BBC3 (PUMA)	ENSG00000105327	−3.66	0.0124	−1.51	0.4215	BCL2 binding component 3
BID	ENSG00000015475	2.50	0.0140	3.12	0.0197	BH3 interacting domain death agonist
BNIP3	ENSG00000176171	5.82	0.0787			BCL2/adenovirus E1B 19 kDa interacting protein 3
BNIP3L	ENSG00000104765			−2.57	0.0034	BCL2/adenovirus E1B 19 kDa interacting protein 3-like
MCL1	ENSG00000143384			1.57	0.0822	Myeloid cell leukemia sequence 1 (BCL2-related)
BOK	ENSG00000176720			−8.21	0.0216	BCL2-related ovarian killer
CARD family						
CARD4	ENSG00000106100	−3.65	0.2323	−1.60	0.5178	NLR family, CARD domain containing 1
CARD6	ENSG00000132357	−8.99	0.0703	−8.26	0.0726	Caspase recruitment domain family, member 6
PYCARD	ENSG00000103490			−1.71	0.1412	PYD and CARD domain containing
NALP1	ENSG00000091592			2.69	0.3319	Pyrin domain containing 1
APAF1	ENSG00000120868	−1.60	0.0869	1.61	0.4612	Apoptotic peptidase activating factor 1
Caspases						
CASP1	ENSG00000137752	3.51	0.0071	3.47	0.3375	Caspase 1, apoptosis-related cysteine peptidase (interleukin 1, beta, convertase)
CASP2	ENSG00000106144	1.55	0.1557			Caspase 2, apoptosis-related cysteine peptidase
CASP3	ENSG00000164305	−1.84	0.0099			Caspase 3, apoptosis-related cysteine peptidase
CASP4	ENSG00000196954	1.84	0.0401			Caspase 4, apoptosis-related cysteine peptidase
CASP6	ENSG00000138794	−4.90	0.0558			Caspase 6, apoptosis-related cysteine peptidase
CASP7	ENSG00000165806			−2.63	0.0039	Caspase 7, apoptosis-related cysteine peptidase
CASP8	ENSG00000064012	5.32	0.0333	2.22	0.0746	Caspase 8, apoptosis-related cysteine peptidase
CASP8AP2	ENSG00000118412			1.91	0.1153	Caspase 8 associated protein 2
CASP9	ENSG00000132906			3.29	0.2507	Caspase 9, apoptosis-related cysteine peptidase
CASP10	ENSG00000003400	−4.50	0.0030	−15.80	0.0018	Caspase 10, apoptosis-related cysteine peptidase
CFLAR	ENSG00000003402	−19.61	0.0354	−5.82	0.0522	CASP8 and FADD-like apoptosis regulator
IAP family						
BIRC2	ENSG00000110330	4.00	0.4295			Baculoviral IAP repeat-containing 2
BIRC3	ENSG00000110330	−2.15	0.5738	2.60	0.1416	Baculoviral IAP repeat-containing 3
BIRC4	ENSG00000110330	−3.11	0.4686			Baculoviral IAP repeat-containing 4
BIRC5	ENSG0000008968	3.50	0.0000	2.70	0.0000	Baculoviral IAP repeat-containing 5
BIRC6	ENSG00000115760			2.09	0.0265	Baculoviral IAP repeat-containing 6
BIRC8	ENSG00000180152			4.13		Baculoviral IAP repeat-containing 8
Others						
HIP1	ENSG00000127946			−1.84	0.0150	Huntingtin interacting protein 1
DAPK1	ENSG00000196730	−27.70	0.0061	−2.08	0.0531	Death-associated protein kinase 1
DEDD	ENSG00000158796	−1.54	0.1765	1.87	0.1560	Death effector domain containing
DIABLO	ENSG00000184047	1.74	0.0082	1.94	0.0181	Homo sapiens diablo homolog (Drosophila)
ESRRBL1	ENSG00000114446			2.11	0.2580	HIP1 protein interactor
HTRA2	ENSG00000115317	1.54	0.2200			HtrA serine peptidase 2

ANOVA analysis with Bonferroni correction was used to determined statistical significance

The anti-apoptotic characteristics of LN18 cells are supported by the downregulation of several genes from the membrane stress receptors, such as TNFRSF1B, TNFRSF10B, TNFRSF10A, along with CFLAR, which is a downstream regulator of caspase 8 activity. In Daoy cells, the regulation of the TNF receptor pathway was not significantly affected, but there was a modest upregulation of NFkB family members (RELB, NFKBIA, NFKBIB). Enhancement of the NFkB signaling pathway suggested a decline in inflammatory processes and strong anti-apoptotic properties for this cell line.

The regulation of both pathways—apoptotic and inflammatory—may subsequently result in the inactivation of certain signal cascades, and ultimately lead to cell survival through their stabilizing effect on the mitochondrial membrane (decrease in Bax, and increase in the Bcl and Bcl-xL families) and downregulation of caspase 10.

### Pathway analysis

Pathway analysis was performed only for genes for which the fold change in their normalized expressions (compared to control HUVECs) reached the significance threshold of <−1.5 or >1.5. MetaCore™ software (from GeneGo; http://www.genego.com/metacore.php) was used for this analysis. ANOVA with Boferroni correction identified statistically significant changes in gene expression, which were visualized on the pathway maps.

### Tumor necrosis factor (TNF) and TNF receptor family pathway

**Fig. 2:**
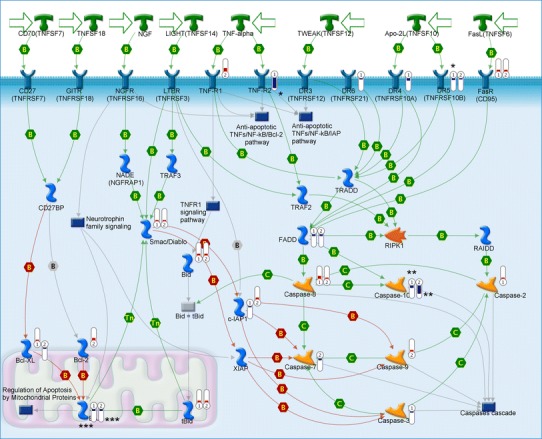
The Metacore™ apoptosis signaling pathway relating to TNF receptors, NFkB and BCL-2 antiapoptotic family genes. *Bars* indicate the degree of up- or downregulation of the gene target relative to HUVEC cells;  means decreased gene expression.  means increased gene expression; height of *bars* corresponds to the change value. *Numbers in symbols* indicate:* 1* LN18,* 2* Daoy cell lines; *number of asterisks* reflects the statistical significance of the *p* value (* *p* < 0.05, ** *p* < 0.01, *** *p* < 0.001)

The expression patterns of the genes that were significantly different in LN18 and Daoy cells compared to reference HUVEC cells reflect the anti-apoptotic properties of the cancer cells as mediated by the TNF receptor family pathway (Fig. 2). In LN18 cells, there was the most pronounced decrease in TNFR2 expression, followed by downregulation of TNFRSR10A (DR4), TNFRSR10B (DR5), TNFRSR21 (DR6), and FADD. There were some less evident changes in TNFR-related gene expression in Daoy cells, in which TNFRSF10B (DR5) and FADD were downregulated while TNFR1 was upregulated.

Only the downregulation of TNFRSF1B (FC = −19.42, *p* = 0.01) and that of TNFRSF10B (FC = −2.73, *p* = 0.01) in LN18 cells were statistically significant (Table 1).

### NFkB signaling pathway

Both cancer cell lines demonstrate altered regulation of the NFkB signaling pathway, since there is generally increased expression of the main component of NFkB complexes: RELA (p65), its inhibitors: KBKE, kinase of inhibitors CHUK, and kappa light polypeptide gene enhancer in B-cells 3, p65: NFKBIA and NFKBIB. In addition, there was increased expression of IKBKG and NFKB1, and a decrease in TBK-1 in the LN18 cell line. In Daoy cells, NFKB2 (p52), REL, RELA (p65), RELB, IKBKE, CHUK, and TBK1, a serine/threonine phosphatase that leads to the activation of NFkB, were all upregulated in comparison to control HUVECs. Protein Bcl-3, the expression of which is upregulated in both cancer cell lines, may bind to NFKB1 and NFKB2 homodimers and form complexes that function as transcriptional activators.

The changes in NFkB pathway gene expression were statistically significant in the group of inducers of inflammatory response. NFKBIA was decreased in LN18 (FC = −1.86, *p* = 0.048) and increased in Daoy cells (FC = 2.11, *p* = 0.0013), IKBKG was increased (FC = 2.3, *p* = 0.0012) in LN18 cells, while RELB (FC = 4.51, *p* = 0.014) and NFKBIB (FC = 2.33, *p* = 0.01) were upregulated in Daoy cells.

### BCL2 family, mitochondrial pathway

**Fig. 3:**
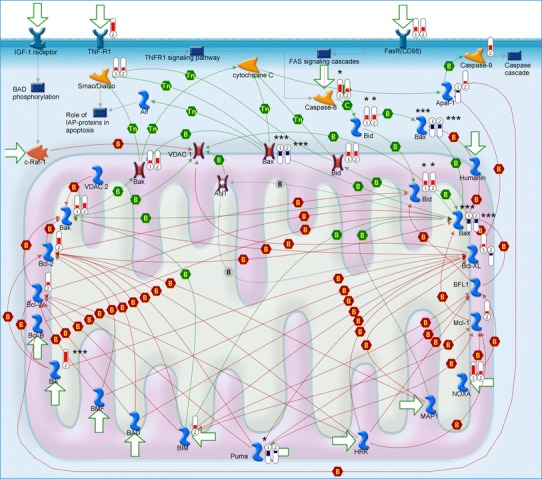
The Metacore™ apoptosis signaling pathway relating to mitochondrial proteins. *Bars* indicate the degree of up- or downregulation of the gene target relative to HUVEC cells;  means decreased gene expression.  means increased gene expression; height of *bars* corresponds to the change value. *Numbers in symbols* indicate:* 1* LN18,* 2* Daoy cell lines; *number of asterisks* reflects the statistical significance of the *p* value (* *p* < 0.05, ** *p* < 0.01, *** *p* < 0.001)

The significantly lower expression of the BAX gene, shown in Fig. 3, may indicate increased anti-apoptotic properties of both Daoy and LN18 cells. Our findings demonstrated increased expression of Bcl-xL and Bcl2 in Daoy cells, in which they serve as protective factors against apoptosis. In both cell lines, the BBC3 (PUMA) and BAX genes were downregulated. There was also a significant decrease in BOK gene expression and moderate downregulation of BNIP3L and BCL2L1 in the Daoy cell line. Genetic analysis also revealed significant upregulation of the PMAIP (NOXA), BNIP3, BID, BAK1, BCL2L11 genes in LN18 and BNIP3, BIK, BCL2, BAK1, BID, PMAIP (NOXA), BCL2L11, and BCL2L2 in Daoy cells.

The most statistically significant changes in the mitochondrial pathway were discovered in BAX (BCL2-associated X protein) gene expression, which was decreased in both LN18 cells (FC = −3.66, *p* = 0.0001) and Daoy cells (FC = −2.78, *p* = 0.0003), and BID, which showed elevated gene expression (LN18, FC = 2.5, *p* = 0.014; Daoy, FC = 3.12, *p* = 0.019). The other factors included PUMA, which was downregulated in LN18 cells, and a group of genes that showed either elevated (BIK) or decreased (BNIP3L, BOK) expression in Daoy cells.

### Caspase family: executive pathway

**Fig. 4:**
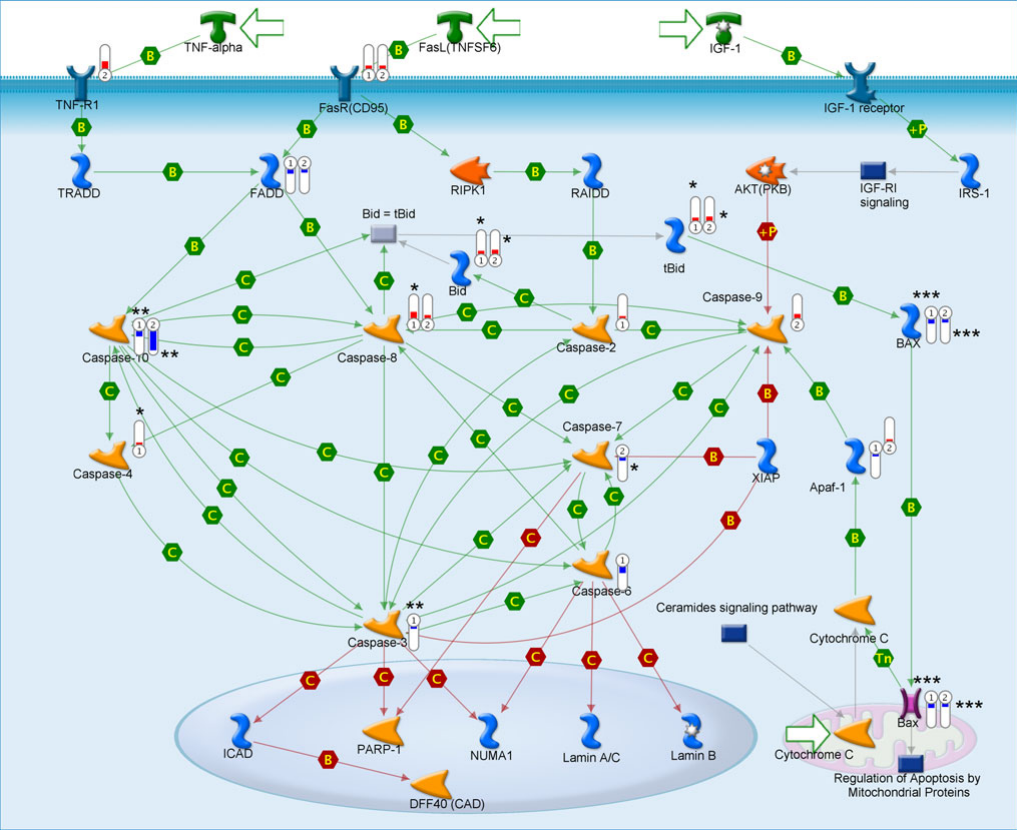
The Metacore™ apoptosis signaling pathway relating to caspase activation. *Bars* indicate the degree of up- or downregulation of the gene target relative to HUVEC cells;  means decreased gene expression.  means increased gene expression; height of *bars* corresponds to the change value. *Numbers in symbols* indicate:* 1* LN18,* 2* Daoy cell lines; *number of asterisks* reflects the statistical significance of the *p* value (* *p* < 0.05, ** *p* < 0.01, *** *p* < 0.001)

Our results show that caspase 4 (also known as caspase 11 or inflammatory caspase) is upregulated in Daoy cells, but it does not seem to play any important causative role in the activation of caspase 3, since the latter is downregulated. Some of the anti-apoptotic effects may be reflected in the downregulation of caspases 7 and 10 in Daoy cells and caspases 6 and 10 in LN18. The expression of the CFLAR gene, which is an apoptosis regulator that may function as a crucial link between cell survival and cell death pathways in mammalian cells, is downregulated in both cancer cell lines (in comparison to HUVECs) (Fig 4; Table 1).

The statistically significant changes in the caspase executive pathway were noted. Most of the caspase gene expression changes were statistically significant in LN18 cells. The CASP3, CASP10, and CFLAR genes were downregulated, while CASP1, CASP4, and CASP8 were slightly increased. The only significant alterations in gene expression were seen in the negative fold changes of the CASP7, CASP10, and CFLAR genes in Daoy cells.

### Inhibitors of apoptosis BIRC proteins

BIRC proteins (baculovirus IAP repeat containing proteins) are members of the inhibitor of apoptosis (IAP) gene family which encode negative regulatory proteins that prevent apoptotic cell death. The proteolytic activity of caspases is tightly controlled by the family of inhibitors of apoptosis (BIRC/IAP), which were typically upregulated in the studied cancer cell lines (Table 1). The gene expression of BIRC2 and BIRC5 was increased in LN18, whereas BIRC3, BIRC5, BIRC6, and BIRC8 were elevated in Daoy cells.

The distinct increase in the fold change in BIRC5 expression was statistically significant in both cancer cell lines compared to the control HUVEC cells. There was also clear upregulation of the BIRC6 gene (FC = 2.09, *p* = 0.026) in Daoy cells.

### CARD family: the caspase recruitment domains

Caspase recruitment domains, or caspase activation and recruitment domains (CARDs), are interaction motifs found in a wide array of proteins, typically those involved in processes relating to inflammation and apoptosis. These domains mediate the formation of larger protein complexes via direct interactions between individual CARDs.

There were several members of the CARD family of genes that were substantially downregulated in the two analyzed cancer lines. Negatively affected genes included CARD4, CARD6, APAF1, and BCL10 in neuronal LN18 cells, and CARD4, CARD6, and PYCARD in the Daoy cell line (Table 1). There was also noticeable but marginal upregulation of NALP1, APAF1, and BCL10 in Daoy cells. None of the observed fold changes in gene expression were statistically significant.

### DED domain genes

DAPK1 (death-associated protein kinase 1) is a multi-domain Ser/Thr protein kinase that plays an important role in apoptosis regulation, and the DEDD death effector domain is a protein–protein interaction domain shared by adaptors, regulators, and executors of the programmed cell death pathway.

DAPK1 was statistically significantly downregulated in both studied cancer cells, in particular LN18 (FC = −27.7, *p* = 0.006). Gene expression of DEDD was modestly decreased in LN18 cells, in contrast to its increase in Daoy cells. There was also slight but statistically significant fold change increases in the expression of DIABLO (Homo sapiens diablo homolog) in both LN18 (FC = 1.74, *p* = 0.008) and Daoy (FC = 1.94, *p* = 0.018) cells.

## Discussion

The two brain cancer cell lines Daoy and LN18 are widely used in many studies of cancerogenesis and apoptosis. The goal of our study was to analyze pro- and anti-apoptotic gene expression using the commercially available set of genes included in the low-density microarray card supplied by Applied Biosystems. The results presented above show the differences between human brain cancer cells and non-cancer cells of endothelial origin.

Current understanding of the molecular phenotypes of medulloblastoma and glioblastoma is still limited, so our gene expression signatures may increase knowledge about the mechanisms of malignant transformation, physiology, and increase the number of potential therapeutic approaches.

Perturbation of apoptosis can lead to excessive cell survival, and can contribute to carcinogenesis [29, 30]. Our study demonstrates the anti-apoptotic properties of both cancer cell lines at the gene expression levels. Such an expression signature is a good mirror of the genetic and epigenetic abnormalities characteristic of the molecular phenotypes of different cell lines.

DAPK1 gene expression was significantly lower in the two studied cancer cell lines in comparison with the non-cancer endothelial cells. The low expression of DAPK1 was regarded as principlally an anti-apoptotic phenomenon, which has been previously observed in many cancer cells [31, 32]. It was hypothesized that Ded protein, a product of DAPK1 gene, might cause cell immortalization and determine malignancy in cancers [32].

LN18 and Daoy cells display anti-apoptotic properties, as exemplified by the regulation of the expression of several apoptotic genes. Different types of human tumors manifest a deregulated NFκB signaling pathway, where NFκB is constitutively active. There was overexpression of many genes which encode proteins that are either members of the NFkB signaling pathway or its regulators. Active NFκB stimulates the expression of the genes that maintain cellular proliferation and protect cells from the conditions that would otherwise cause their apoptosis [1–3].

Our results demonstrate a number of statistically significant changes in the genes related to any of the aspects of the apoptotic pathway. In addition to the genes listed above, the most pronounced fold changes in gene expression were noted in CASP10, CFLAR, BAX, and BID. LN18 cells differed from the Daoy cell line in their more pronounced regulation of the genes encoding the TNF receptor pathway and caspase family. Such a gene expression pattern might provide the neuronal LN18 cells with an advantageous control over inflammatory processes as well.

The dynamic balance between relative amounts of pro- and anti-apoptotic proteins is believed to be the key determinant in the regulation of cell death and survival [33–35]. Thus, the presented differences in basal condition gene expression should be considered when either LN18 or Daoy cell lines are selected as models in molecular structural and/or functional studies of other factors.

We showed in our work that endothelial cells are more susceptible to apoptosis than glioblastoma and medulloblastoma. Endothelial cells (ECs) are also ubiquitous within tumors because tumors are vascular, and yet, the impact of tumor-resident ECs is less well understood. Radical therapy, which is usually used in the treatment of many kinds of tumors due to its pro-apoptotic properties, may influence not only tumor cells but also first line endothelial cells, inhibiting its influence on cancer growth. Cancer growth and metastasis are regulated in part by stromal cells such as fibroblasts and immune cells within the tumor microenvironment. In our work, HUVEC cells were used as a model of endothelial cells. Endothelial cells play a crucial role in tumor angiogenesis, which is necessary for solid tumor progression and metastasis. Through paracrine regulation, ECs modulate a diverse spectrum of pathophysiologic processes in normal and hyperplastic tissues. ECs offer similar paracrine regulatory control of cancer biology. Indeed, secretions from quiescent ECs muted the proliferative and invasive phenotype of lung and breast cancer cells in vitro, and reduced cancer cell protumorigenic and proinflammatory signaling. EC perlecan silencing significantly changed this regulatory relationship, eliminating the ability of ECs to inhibit cancer cell invasiveness via increased interleukin-6 secretion. Moreover, implanting ECs embedded within porous matrices slowed adjacent xenograft tumor growth and prevented architectural degeneration, concomitantly reducing proliferative and tumorigenic markers. Finally, lung carcinoma cells pretreated with intact EC-conditioned media, but not media conditioned with perlecan-silenced ECs, exhibited a reduced micrometastatic burden after tail vein injection [37]. These findings add to an emerging appreciation of EC-regulatory effects that transcend their structural roles and pave the way for improved characterization and control of EC–cancer cross-talk interactions for the diagnosis, prognosis, and treatment of cancer.
